# Comparative Studies of Inhibitory and Antioxidant Activities, and Organic Acids Compositions of Postbiotics Produced by Probiotic *Lactiplantibacillus plantarum* Strains Isolated From Malaysian Foods

**DOI:** 10.3389/fvets.2020.602280

**Published:** 2021-01-26

**Authors:** Hui Mei Chang, Hooi Ling Foo, Teck Chwen Loh, Eric Teik Chung Lim, Nur Elina Abdul Mutalib

**Affiliations:** ^1^Department of Animal Science, Faculty of Agriculture, Universiti Putra Malaysia, Selangor, Malaysia; ^2^Department of Bioprocess Technology, Faculty of Biotechnology and Biomolecular Sciences, Universiti Putra Malaysia, Selangor, Malaysia; ^3^Institute of Bioscience, Universiti Putra Malaysia, Selangor, Malaysia; ^4^Institute of Tropical Agriculture and Food Security, Universiti Putra Malaysia, Selangor, Malaysia; ^5^Agro-Biotechnology Institute (ABI), National Institute Biotechnology Malaysia (NIBM), Selangor, Malaysia

**Keywords:** *Lactiplantibacillus plantarum*, postbiotic, natural bioactive ingredients, organic acids, antimicrobial, antioxidant, anti-inflammatory

## Abstract

Despite inflammation being a protective natural defense against imbalance stressors in the body, chronic inflammation could lead to the deterioration of immune response, low production, and poor performance in livestock as well as severe economic losses to the farmers. Postbiotics produced by *Lactiplantibacillus plantarum* has been reported recently to be a natural source of antioxidant, promoting growth performance, anti-inflammation, and immune responses. However, the effects of fermentation media on the compositions of *L. plantarum* postbiotic have not been reported elsewhere. Hence, a comparative study was conducted to compare the volatile compounds, organic acid composition, and antioxidant and antimicrobial activities of postbiotics produced by six strains of *L. plantarum* cultivated by using formulated media and the commercial de Man, Rogosa, and Sharpe (MRS) medium as a control. Postbiotics RG14, RI11, and UL4 produced by using formulated media exhibited higher inhibitory activity against *Pediococcus acidilactici* 446, *Escherichia coli* E-30, *Salmonella enterica* CS3, and vancomycin-resistant *Enterococci* except for *Listeria monocytogenes* LS55. As for the antioxidant activity, hydroxyl radical scavenging activity was enhanced in formulated media, whereas reducing power activity was the highest in postbiotic RI11. Three organic acids, namely, acetic acid, caproic acid, and lactic acid, were detected in the postbiotic produced by various *L. plantarum* strains. The concentration of acetic acid was influenced by the fermentation media, whereas caproic acid was detected as the highest in postbiotic RG11. Lactic acid was the predominant compound detected in all the postbiotics and had the significantly highest concentration in postbiotic RS5 when produced by using the MRS medium. Intermediary and pyrrole compounds were the other main compounds that were detected by using GC-MS. Positive correlations were found between organic acid production and inhibitory activity, as well as antioxidant activity exhibited by postbiotics. In conclusion, the compositions and functional characteristics of postbiotics produced by the six strains of *L. plantarum* were strain-dependent and affected greatly by the fermentation medium. The effects of postbiotic composition on the functional characteristics of postbiotics were elucidated in this study to warrant their applications as a promising beneficial natural growth promoter for the livestock industry.

## Introduction

Inflammation is an essential natural defense process that occurs when there is an imbalance of stressors caused by pathogens, allergens, radiation, toxin, and oxidative stress in the body (1). As a series of protective mechanisms involving the molecular reactions and cellular activity, inflammation speeds up tissue repair to prevent permanent damage (2–4). The inflammatory process also helps to build immunity against inevitable microbial challenges such as *Salmonella* sp. and *Eimeria* sp. infections by stimulating rapid expression of pro-inflammatory cytokines, maintains homeostasis, and improves vaccine efficacy (5, 6), and hence enhances the resistance of diseases in commercially farmed animals.

Although inflammation is a fundamental process to fight infection, prolonged and chronic inflammation leads to damaging consequences such as tissue and organ damage, and mortality (2). In livestock farming, numerous factors induce the occurrence of inflammation, including the intensification of production, poor quality, and contamination in feedstuffs as well as genetic selection (7). As a result, this will contribute to poor growth performance and production due to low nutrient absorption, loss of appetite, impaired intestinal architecture, and leaky gut (4, 8), which lead to a large amount of energy consumption on the immune system instead of growing and production (9). Consequently, inflammation reduces the economic viability of farmers and food product quality, and endangers animal health. To encounter this phenomenon, natural bioactive ingredients synthesized by probiotic bacteria serve as potential regulators of inflammation (10, 11).

*Lactiplantibacillus plantarum* (*L. plantarum*) or previously known as *Lactobacillus plantarum* (12) has been shown to synthesize various beneficial and economically important extracellular metabolites known as postbiotic, which contains organic acids such as lactic acid and other short-chain fatty acids (SCFAs). Unlike previously reported pre-, pro-, and paraprobiotic, postbiotic is a bioactive soluble compound or peptide that is produced from complex metabolic fermentation activity during the growth of lactic acid bacteria (13, 14). Postbiotics confer health benefits on the host or food (15) to prevent infection, antitumor, immunomodulatory, and antiatherosclerotic effects and improved wound healing (16), as compared to cell-free supernatant preparations. Moreover, postbiotics are well documented for their anti-inflammatory and broad spectrum of antimicrobial activities (13, 17, 18). Other compounds that are secreted along with organic acids include vitamins, enzymes, extracellular proteins, indole, immune signaling compounds, cofactors, extracellular vesicles, and complex agents that are involved in regulating the intestinal epithelial barrier to promote better gut health (19). The antimicrobial peptides of bacteriocin molecule present in the postbiotic function as an antimicrobial agent against various pathogens, i.e., *Salmonella* sp. and *Escherichia coli* (20–22). Furthermore, supplementation of *L. plantarum* postbiotics in feed improves the growth performance of livestock (23, 24), laying performance (25), villus height (25, 26), antioxidant activity in the blood (27), and immune response (24), and reduces adverse impacts on the physiological activities of broilers due to heat stress (28). However, the production of postbiotic metabolites is affected by various factors such as temperature and pH (29), strains of producer bacteria, and nutrient components of the growth medium (30, 31).

Bacterial fermentation technology has gained vast attention as it produces compounds that have economic value for various applications such as biofuel, pharmaceutical products, and chemicals for various industries (32). Bacteria require sufficient carbon, nitrogen, and other nutrients in the growth medium to improve growth, metabolic activity, and production of vital metabolites (33). As reported previously, the optimization of fermentation media enhanced the bacteriocin inhibitory of postbiotic produced by *L. plantarum* I-UL4 (31). Moreover, *L. plantarum* postbiotics exerted higher inhibitory activity against various pathogenic bacteria such as *E. coli, Salmonella enterica, Listeria monocytogenes*, and VRE when inulin is included in the fermentation media (22). This further promotes the growth performance, growth-related gene expression, meat quality, and fecal microbiota in broiler chickens (21, 34). From the studies, fermentation media have been reported to affect the secretion of metabolic products and biological activities of postbiotic.

Although there are extensive reports on the benefits of *L. plantarum* postbiotic as a natural growth promoter to replace antibiotic growth promoter (AGP), the link between formulated media and the composition, antioxidant activity, and antimicrobial activity of postbiotics is yet to be explored. Therefore, the objective of this current research was to compare the composition and functional characteristics of postbiotics produced by six strains of *L. plantarum* using different formulated media. The effect of postbiotic composition on the functional characteristics of postbiotics would be elucidated in this study to warrant their applications as a promising beneficial natural growth promoter for the livestock industry.

## Materials and Methods

### Bacteria Strains and Maintenance

The six strains of *L. plantarum* that were used in this study, namely, RG11, RG14, RI11, RS5, TL1, and UL4, which were previously isolated from Malaysian fermented foods (35, 36), and obtained from the Laboratory of Industrial Biotechnology, Department of Bioprocess Technology, Faculty of Biotechnology and Biomolecular Sciences, Universiti Putra Malaysia. The *L. plantarum* cultures were maintained and revived as described by Foo et al. (37). *Pediococcus acidilactici* 446, which was employed as a positive indicator bacterium, was cultured in an MRS medium, while the pathogenic bacteria *E. coli* E-30 and *S. enterica* CS3 were propagated in a Luria Bertani (LB) medium. *L. monocytogenes* LS55 was cultured in Listeria enrichment broth (LEB), and vancomycin-resistant *Enterococci* was cultivated in a brain heart infusion (BHI) medium. All the pathogens were revived twice for 48 h and 24 h at 37°C with agitation at 150 rpm using an incubator shaker according to the method described by Kareem et al. (22).

### Preparation of Postbiotic

The active *L. plantarum* strains were washed once with sterile 0.85% (w/v) NaCl (Merck, Darmstadt, Germany) solution and adjusted to 10^9^ CFU/mL to be used as a 10% (v/v) inoculum according to the method described by Mohamad Zabidi et al. (38). The postbiotics of the six strains of *L. plantarum* were prepared at 30°C for 24 h according to the method described by Kareem et al. (22) by using MRS as a control medium and their respective formulated media as shown in Table 1. The postbiotics were harvested after centrifugation at 10,000 × g for 15 min at 4°C and filtered through a 0.22 μm cellulose acetate membrane (Sartorius Minisart, Germany) to remove the residue of viable producer cells.

**Table 1:** Medium composition of de Man, Rogosa, and Sharpe and formulated media for postbiotic production by six *L. plantarum* strains.

**Medium composition (g/L)**	**MRS**	**RG11**	**RG14**	**RI11**	**RS5**	**TL1**	**UL4**
Glucose	20.0	20.0	20.0	20.0	20.0	20.0	20.0
Yeast extract	4.00	27.8	27.8	27.8	27.8	27.8	36.2
Peptone from casein	10.0	–	–	–	–	–	–
Meat extract	8.00	–	–	–	–	–	–
Dipotassium hydrogen phosphate	2.00	–	–	2.00	–	–	–
Sodium acetate	5.00	4.48	4.00	5.00	5.75	4.88	3.70
Magnesium sulfate	0.20	0.30	–	0.20	–	–	–
Manganese sulfate	0.04	–	–	–	–	–	–
Manganese sulfate tetrahydrate	–	0.06	0.03	0.04	0.05	0.04	0.03
Tween 80	1.00	1.19	1.00	1.00	1.12	1.01	0.76
Diammonium hydrogen citrate	2.00	–	1.50	2.00	–	–	–

### Inhibitory Activity by Agar Well Diffusion Assay

The postbiotics of all *L. plantarum* strains were diluted through two-fold serial dilution using NaCl solution (0.85% w/v). The diluted postbiotics were loaded at 20 μ*L* for *P. acidilactici* 446 and 100 μ*L* for *E. coli* E-30, *S. enterica* CS3, *L. monocytogenes* LS55, and VRE, respectively, into the pre-punched agar well according to the method described by Kareem et al. (22). All the plates were allowed to dry at room temperature for about 30–60 min. Then, the agar plates were overlaid with soft agar seeded with the respective bacterial cells before incubation at 37°C for 24–48 h. A clear inhibition zone with more than 0.1 cm formed around the well was measured and recorded as a positive inhibitory result. The experiment was conducted with three replicates. The inhibitory activity was determined as a modified arbitrary unit (MAU/ml) (31) by using the following formula:

$$\begin{array}{l} \text{Modified arbitrary unit }\left(\text{MAU}/\text{mL}\right)\\ =\frac{\text{Highest dilution yield of inhibition zone }\left(\text{AU}\right)}{\text{Volume of postbiotic }\left(\text{mL}\right)\;\times \text{Diameter of inhibition zone }\left(\text{cm}\right)} \end{array}$$

### Determination of Antioxidant Activity

#### Hydroxyl Radical Scavenging Assay

The hydroxyl radical scavenging (HRS) activity was determined by following the method of Xing et al. (39) with slight modification. Each 250 μL sample was added with 250 μL of 2.5 mM 1,10-phenanthroline, 250 μL of PBS (0.1 M, pH 7.4), and 250 μL of 2.5 mM FeSO_4_ prior mixed with 250 μL of 20 mM H_2_O_2_. The test solution was incubated at 37°C for 90 min and absorbance was read at 536 nm using a UV spectrophotometer. HRS activity was determined using the following equation:

$$\begin{array}{l} \text{HRS activity }\left(\text{\%}\right)=\left({\text{A}}_{\text{s}}-{\text{A}}_{\text{c}}\right)/\left({\text{A}}_{\text{b}}-{\text{A}}_{\text{c}}\right)\times 100\% \end{array}$$

where A_s_ is the absorbance of the sample, A_c_ is the absorbance containing the deionized water, and A_b_ is the absorbance of the solution without the presence of the sample and H_2_O_2_. All the samples were tested with three replications.

#### Reducing Power

The reducing power (RP) of the postbiotics was determined using the method of Xing et al. (39) with slight modification. The assay mixture contained 250 μL of the sample, 250 μL of PBS (0.2 M, pH 6.6), and 250 μL of 1% potassium ferricyanide before heating at 50°C for 20 min. The test solution was allowed to cool down to room temperature. Then, the mixture was added with 250 μL 10% trichloroacetic acid, centrifuged at a speed of 3,000 × g for 5 min at 4°C. The supernatant was collected and added with 250 μL deionized water. Absorbance was taken at 700 nm after adding 500 μL 0.1% iron (III) chloride. Ascorbic acid was used as the standard. All the samples were repeated in triplicates.

#### Quantification of Short-Chain Fatty Acids in Postbiotics

The SCFAs in postbiotics were determined by using gas chromatography according to Nakkarach et al. (40) with slight modifications. A 500 μL postbiotic produced by each strain was added with 500 μL 5 mM 4-methyl valeric acid, and 1 μL of the sample was injected into a gas chromatograph (GC, Agilent Technologies, USA). The GC was equipped with a flame ionization detector and a DB-FFAP 122-3232 fused-silica capillary column (30 m × 0.25 μm × 0.25 μm). The temperature of the column was set at 100°C, increased to 180°C with 10°C/min for accurate quantification. Temperatures for the detector, inlet, and oven were set at 250°C, 230°C, and 230°C, respectively. Nitrogen gas was used as the carrier gas with a flow of 35 mL/min, while methanol was used as the solvent. Acetic acid (10 mM), propionic acid (10 mM), butyric acid (10 mM), isobutyric acid (10 mM), isovaleric acid (10 mM), valeric acid (10 mM), caproic acid (10 mM), and 4-methyl valeric acid (5 mM) were used as standard solutions to identify the peaks. All the samples were tested in triplicates. The calculation of the acid concentration was done by using the following formula:

$$\begin{array}{l} \text{Volatile fatty acid concentration }\left(\text{mM}\right)=\\ \frac{\text{a}}{2\text{ mv}}\times \frac{\text{Concentration of VFA in standard mixture }\times 2}{\text{A}/2\text{ MV}} \end{array}$$

where A, 2 MV = peak heights of VFA and 4-methyl valeric acid in the standard mixture

a, and 2 mv = peak heights of VFA and 4-methyl valeric acid in a sample.

### Determination of Lactic Acid

The lactic acid concentration (g/L) in postbiotics was determined according to Borshchevskaya et al. (41). Briefly the postbiotic was diluted using pure water before being added with 2 mL 0.2% iron (III) chloride. The absorbance of the test solutions was read at 390 nm using a UV-visible spectrophotometer (Agilent Technologies, USA) before 15 min upon mixing. A standard curve was plotted using standard lactic acid (L6661, Sigma Chemical, USA) at the concentration of 0–80 mg/mL. All the samples were tested with three replications.

#### Identification of Volatile Compounds Using Gas Chromatography–Mass Spectrometry (GC–MS) Analysis

The samples were prepared according to Kam et al. (42) with slight modifications. An equal volume of ethyl acetate (HPLC grade) was used to extract 30 mL of supernatants twice. The mixture of supernatant and ethyl acetate was stirred for about 15–20 min before allowing the separation into water and organic phase by using a separatory funnel. The collected water phase was allowed to re-extract again with ethyl acetate, while the organic phase was transferred into a Schott bottle. The organic phase produced from the second extraction was combined with the previously collected organic phase. Then, the organic phase was allowed to dry up by using a rotary evaporator (Buchi, Switzerland) at 40°C before being added with 500 μL of methanol (HPLC grade). The mixture was then allowed to dry in a fume chamber overnight (12–15 h) at room temperature. All the samples were added with 1 mL of methanol before being filtered with a syringe filter of 0.2 μ*m*. The samples were analyzed using GC–MS (Shimadzu model GC-2010) connected with QP2010 Ultra as the analytical line. A column of RXI-5MS (30 m × 0.25 mm × 0.25 μm) was used with the injector temperature at 250°C in split mode. Helium was used as the carrier gas. The oven temperature was set at 50°C and was increased at 3°C/min to 300°C and held for 10 min. For the analytical part, the ion source was set at 200°C, EM voltage at 0.92 kV, and solvent cut time at 2 min. Scan mode was used and performed from the range of 40 to 700 m/z starting from 2.4 to 93.0 min. The identification of compounds was based on NIST Library 11 and Willey 229.

#### Statistical Analysis

The collected data except for GC-MS analysis were analyzed using two-way analysis of variance (ANOVA) using the Statistical Analysis System (SAS) program (version 9.4) to determine the significant difference and interactions among the factors. Tukey's Honest Significant Difference Test was used to compare a significant difference of treatment means at *P* < 0.05. A correlation test was used to determine the relationship between the compositions of the postbiotics and the functional characteristics.

## Results

### Inhibitory Activity of Postbiotics

The inhibitory activities exhibited by each postbiotic against a positive indicator and pathogenic bacteria are shown in Table 2. Generally, the modified inhibitory activity of *L. plantarum* postbiotics was enhanced (*P* < 0.05) when produced by using formulated media except for the modified inhibitory activity against *L. monocytogenes* LS55. Postbiotic RG14 exhibited the highest (*P* < 0.05) inhibitory activity against a positive indicator, *P. acidilactici* 446. Moreover, the postbiotic RG14, RS5, and TL1 produced significantly (*P* < 0.05) stronger antimicrobial action for *E. coli* E-30, as compared to other strains of *L. plantarum*. Besides, postbiotics RG14 and RI11 had higher (*P* < 0.05) inhibitory activity against *S. enterica* CS3 in comparison to other postbiotics. Postbiotic RI11 and UL4 produced the highest and lowest (*P* < 0.05) inhibitory activities against VRE, whereas postbiotic RS5 exhibited the lowest (*P* < 0.05) inhibitory activity when tested against *L. monocytogenes* LS55, even though it showed no significant difference (*P* > 0.05) with RG11 and RG14.

**Table 2:** Inhibitory activity of *L. plantarum* postbiotics against positive indicator *P. acidilactici* 446, and pathogenic bacteria *E. coli* E-30, *S. enterica* CS3, VRE, and *L. monocytogenes* LS55.

**Inhibitory activity of postbiotic (MAU/mL)**										
**Postbiotics**	***P. acidilactici*** **446**		***E. coli*** **E-30**		***S. enterica*** **CS3**		***L. monocytogenes*** **LS55**		**VRE**	
	**Control^**1**^**	**FRM^**2**^**	**Control^**1**^**	**FRM^**2**^**	**Control^**1**^**	**FRM^**2**^**	**Control^**1**^**	**FRM^**2**^**	**Control^**1**^**	**FRM^**2**^**
RG11	533 ± 26.7^Bc^	1120 ± 160^Ac^	26.7 ± 1.33^Ba^	28.0 ± 0^Aa^	22 ± 0^Bbc^	22.7 ± 0.67^Abc^	36.0 ± 2.31^ab^	40.0 ± 2.31^ab^	45.3 ± 1.33^Bab^	46.7 ± 1.33^Aab^
RG14	1440 ± 0^Ba^	1813 ± 86.7^Aa^	28.0 ± 0^Ba^	33.3 ± 1.33^Aa^	20 ± 0^Ba^	37.3 ± 1.33^Aa^	32 ± 0^ab^	42.7 ± 1.33^ab^	38.7 ± 1.33^Bbc^	42.7 ± 1.33^Abc^
RI11	1120 ± 0.^Bb^	1440 ± 92.4^ab^	15.3 ± 0.67^Bb^	30.7 ± 1.33^ab^	28 ± 0^Ba^	31.3 ± 1.76^Aa^	41.3 ± 1.9^a^	41.3 ± 2.67^a^	46.7 ± 2.66^Ba^	48.0 ± 2.31^Aa^
RS5	667 ± 26.7^Bd^	587 ± 26.7^Ad^	29.3 ± 0^Ba^	32.0 ± 0^Aa^	20.7 ± 1.44^Bbc^	24.7 ± 1.76^Abc^	32 ± 2.31^b^	33.3 ± 1.33^b^	37.3 ± 4.81^Bbc^	41.3 ± 1.33^Abc^
TL1	1120 ± 0^Bb^	1333 ± 107^ab^	28.0 ± 2.31^Ba^	26.7 ± 1.33^Aa^	20 ± 1.15^Bb^	30.7 ± 0.67^ab^	46.7 ± 5.33^a^	37.3 ± 3.53^a^	42.7 ± 1.33^Babc^	40.0 ± 2.31^Aabc^
UL4	1173 ± 53.3^Bb^	1226 ± 53.3^ab^	14.0 ± 1.15^Bb^	29.3 ± 1.33^ab^	18.9 ± 0.67^Bc^	24 ± 0^Ac^	40 ± 1.33^a^	45.3 ± 3.53^a^	30.7 ± 2.66^Bc^	46.7 ± 1.33^Ac^
*P*-value	<0.0001		<0.0001		<0.0001		0.0046		0.0002	
Media × strains	0.0001		<0.0001		0.001		0.027		0.0031	
Media	<0.0001		<0.0001		0.001		0.25		0.0037	
Strains	<0.0001		<0.0001		<0.0001		0.005		0.0011	

ABabcd Means (mean of modified bacteriocin activity ± SEM) within the same row and column have significant differences at P < 0.05.

1 Control: de-Mann Rogosa Sharpe (MRS) medium.

2 FRM, formulated media.

AB Fermentation media.

*abcd Bacteria strain*.

### Antioxidant Activity of Postbiotics

The HRS activity was significantly higher (*P* < 0.05) for the postbiotics produced by the formulated media as compared to the control MRS medium, even though there was no significant difference among all the producer strains (Table 3). There was significant (*P* < 0.05) interaction between media and strains on the HRS activity. As for the RP activity, among all the postbiotics, RI11 was observed to have the highest (*P* < 0.05) activity but showed no significant difference (*P* > 0.05) with RG14 and UL4 (Table 3). However, there was no significant difference (*P* > 0.05) in the fermentation media used to produce postbiotics on RP activity. There was also no significant (*P* > 0.05) interaction found between media and strains on RP activity.

**Table 3:** Hydroxyl radical scavenging and reducing power activity of *L. plantarum* postbiotics produced by using different fermentation media.

**Postbiotics**	**HRS**		**RP**	
	**Control**^**1**^	**FRM**^**2**^	**Control**^**1**^	**FRM**^**2**^
RG11	30.3 ± 0.2^B^	34.4 ± 0.2^A^	1.97 ± 0.1^b^	1.90 ± 0.1^b^
RG14	30.1 ± 0.3^B^	33.8 ± 0.7^A^	2.23 ± 0.1^ab^	1.97 ± 0.1^ab^
RI11	32.9 ± 0.3^B^	31.9 ± 0.8^A^	2.22 ± 0.1^a^	2.25 ± 0.1^a^
RS5	29.7 ± 0.2^B^	36.2 ± 0.2^A^	2.04 ± 0.1^b^	2.00 ± 0.1^b^
TL1	29.5 ± 0.4^B^	35.3 ± 0.7^A^	2.08 ± 0.1^b^	1.89 ± 0.1^b^
UL4	30.3 ± 0.1^B^	36.9 ± 0.3^A^	2.05 ± 0.1^ab^	2.16 ± 0.1^ab^
*P*-value	<0.001		0.003	
Media × strains	0.01		0.08	
Media	<0.001		0.07	
Strains	0.623		0.002	

^ABabcd^ Means (mean of concentration ± SEM) within the same row and column have significant differences at P < 0.05.

1 Control: de-Mann Rogosa Sharpe (MRS) medium.

2 FRM, Formulated media.

^AB^ Fermentation media.

ab *Bacteria strain*.

### Organic Acids Production

The production of acetic acid was detected in all the *L. plantarum* strains. The highest concentration (*P* < 0.05) of acetic acid was recorded by postbiotic TL1 and UL4, even though UL4 had no significant (*P* > 0.05) difference among other postbiotics (Table 4). There was no significant difference (*P* > 0.05) detected on the media used to produce acetic acid. In comparison, postbiotic RG11 produced the highest caproic acid, while the lowest caproic acid production was detected in TL1 and UL4 (Table 4). On the other hand, the formulated fermentation media had significantly increased (*P* < 0.05) the production of caproic acid in postbiotic metabolite.

**Table 4:** Acetic, caproic, and lactic acid concentration of *L. plantarum* postbiotics produced by using different fermentation media.

**Postbiotics**	**Acetic acid (mM)**		**Caproic acid (mM)**		**Lactic acid (g/L)**	
	**Control^**1**^**	**FRM^**2**^**	**Control^**1**^**	**FRM^**2**^**	**Control^**1**^**	**FRM^**2**^**
RG11	17.3 ± 0.74^b^	17.5 ± 1.51^b^	22.4 ± 1.6^Ba^	23.8 ± 1.04^Aa^	31.8 ± 0.33^Ad^	30.8 ± 0.61^Bd^
RG14	18.5 ± 1.37^b^	16.7 ± 1.72^b^	14.1 ± 2.6^Bbc^	14.9 ± 1.11^Abc^	30.2 ± 1.03^Ad^	30.2 ± 1.03^Bd^
RI11	15.8 ± 0.31^b^	15.4 ± 0.49^b^	8.4 ± 0.57^Bc^	15.1 ± 1.18^Ac^	42.9 ± 0.33^Ab^	35.2 ± 0.6^Bb^
RS5	15.4 ± 1.91^b^	17.8 ± 0.28^b^	17.2 ± 1.63^Bb^	18.7 ± 0.5^Ab^	44.7 ± 0.65^Aa^	42.6 ± 1.31^Ba^
TL1	32.1 ± 0.43^a^	35.1 ± 1.91^a^	5.1 ± 0.2^Bd^	6.32 ± 1.43^Ad^	28 ± 0.37^Ad^	30.4 ± 0.76^Bd^
UL4	33.5 ± 1.56^Ab^	31 ± 1.94^Ab^	4.05 ± 0.29^Bd^	4.29 ± 0.33^Ad^	37.6 ± 1.29^Ac^	31.9 ± 0.84^Bc^
*P*-value	0.03		<0.001		<0.001	
Media × strains	0.74		0.013		<0.001	
Media	0.46		0.01		<0.001	
Strains	0.003		<0.001		<0.001	

^ABabcd^Means (mean of concentration ± SEM) within the same row and column have significant differences at P < 0.05.

1 Control: de-Mann Rogosa Sharpe (MRS) medium.

2 FRM, Formulated media.

AB Fermentation media.

*^abcd^Bacteria strain*.

As the major acid secreted by lactic acid bacteria, postbiotic RS5 produced the highest (*P* < 0.05) concentration of lactic acid (Table 4). Interestingly, the commercial control MRS medium exhibited a greater effect (*P* < 0.05) on the formation of lactic acid, even though postbiotic TL1 that grown in a commercial control MRS medium produced the significantly lowest (*P* < 0.05) yield of lactic acid.

### Identification of Volatile Compounds in Postbiotics Using GC-MS

The volatile compound profiles of postbiotics are shown in Table 5. Altogether, there were 16 organic compounds including ester, acids, and pyrrole compounds that were detected from the postbiotics produced by various strains of *L. plantarum* using GC-MS. Postbiotic RI11 produced by using formulated media contained the highest numbers of volatile compounds, unlike other postbiotics that contained only six types of volatile compounds. Nevertheless, there were 10 volatile compounds identified from postbiotic UL4 produced by using a control MRS medium, 9 volatile compounds from the postbiotics RI11 and RS5, 8 volatile compounds from the postbiotics RG11 and RG14, and 7 volatile compounds from the postbiotic TL1. The relative peak area of lactic acid, which was found to be predominant in all postbiotics (70.65%–93.41%), was noted higher in postbiotic produced by using formulated media in comparison to those postbiotics produced by using a control MRS medium (14.52–70.87%). Moreover, pentanoic acid, 2-hydroxy-4-methyl-, methyl ester, and 1,4-diaza-2,5-dioxo-3-isobutyl bicycle [4.3.0] nonane were detected at the least level in the postbiotics produced by various strains of *L. plantarum*.

**Table 5:** Compounds present in *L. plantarum* postbiotics identified by GC-MS analysis.

**No**.	**Retention time**	**Compounds**	**Peak area (%)**
**Postbiotic RG11 fermented in control medium**			
1.	3.09	Acetoin	7.79
2.	3.47	Propanoic acid, 2-hydroxy-methyl ester	51.56
3.	8.63	Lactic acid	21.53
4.	10.24	Pentanoic acid, 2-hydroxy-4-methyl-, methyl ester	1.43
5.	17.52	2,3-dihydro-3,5-dihydroxy-6-methyl-4H-Pyran-4-one	3.76
6.	48.92	1,4-diaza-2,5-dioxo-3-isobutyl bicyclo [4.3.0] nonane	3.37
7.	49.53	Pyrrolo [1,2-a] pyrazine-1,4-dione, hexahydro-3-(2-methylpropyl)	3.14
8.	62.30	Pyrrolo [1,2-a] pyrazine-1,4-dione, hexahydro-3-(phenylmethyl)	11.40
**Postbiotic RG11 fermented in formulated medium**			
1.	3.10	Acetoin	1.65
2.	3.46	Propanoic acid, 2-hydroxy-methyl ester	2.63
3.	7.98	Lactic acid	92.68
4.	17.52	2-3-dihydro-3,5-dihydroxy-6-methyl-4H-Pyran-4-one	1.13
5.	48.92	1,4-diaza-2,5-dioxo-3-isobutyl bicycle [4.3.0] nonane	1.17
6.	49.92	Pyrrolo[1,2-a] pyrazine-1,4-dione, hexahydro-3-(2-methylpropyl)	0.75
**Postbiotic RG14 fermented in control medium**			
1.	3.10	Acetoin	3.62
2.	3.47	Propanoic acid, 2-hydroxy-methyl ester	51.87
3.	9.17	Lactic acid	14.52
4.	17.06	2-hydroxy-4-methylpentanoic acid	6.41
5.	23.67	2-3-dihydro-3,5-dihydroxy-6-methyl-4H-Pyran-4-one	5.68
6.	48.97	1,4-diaza-2,5-dioxo-3-isobutyl bicycle [4.3.0] nonane	3.35
7.	48.92	Pyrrolo[1,2-a] pyrazine-1,4-dione, hexahydro-3-(2-methylpropyl)	4.00
8.	63.30	Pyrrolo [1,2-a] pyrazine-1,4-dione, hexahydro-3-(phenylmethyl)	10.55
**Postbiotic RG14 fermented in formulated medium**			
1.	3.10	Acetoin	1.24
2.	3.46	Propanoic acid, 2-hydroxy-methyl ester	2.14
3.	7.98	Lactic acid	93.41
4.	17.27	2-3-dihydro-3,5-dihydroxy-6-methyl-4H-Pyran-4-one	1.12
5.	48.89	1,4-diaza-2,5-dioxo-3-isobutyl bicycle [4.3.0] nonane	1.16
6.	48.92	Pyrrolo[1,2-a] pyrazine-1,4-dione, hexahydro-3-(2-methylpropyl)	0.93
**Postbiotic RI11 fermented in control medium**			
1.	3.09	Acetoin	3.49
2.	3.45	Propanoic acid, 2-hydroxy-methyl ester	10.37
3.	7.94	Lactic acid	70.87
4.	10.24	Pentanoic acid, 2-hydroxy-4-methyl-, methyl ester	0.67
5.	17.27	2-hydroxy-4-methylpentanoic acid	3.94
6.	23.67	2-3-dihydro-3,5-dihydroxy-6-methyl-4H-Pyran-4-one	3.18
7.	48.85	1,4-diaza-2,5-dioxo-3-isobutyl bicycle [4.3.0] nonane	1.43
8.	48.92	Pyrrolo[1,2-a] pyrazine-1,4-dione, hexahydro-3-(2-methylpropyl)	1.65
9.	62.30	Pyrrolo [1,2-a] pyrazine-1,4-dione, hexahydro-3-(phenylmethyl)	4.40
**Postbiotic RI11 fermented in formulated medium**			
1.	3.11	Acetoin	1.03
2.	3.46	Propanoic acid, 2-hydroxy-methyl ester	0.77
3.	3.64	Isobutyric acid	0.33
4.	5.23	Butyric acid	0.23
5.	7.98	Lactic acid	70.65
6.	9.67	Pentanoic acid, 2-hydroxy-4-methyl-, methyl ester	0.54
7.	17.21	2-3-dihydro-3,5-dihydroxy-6-methyl-4H-Pyran-4-one	8.17
8.	23.74	2-5-Dimethyl-2,4-dihydroxy-3 (2H)-thiophenone	1.25
9.	33.90	Phenol, 2-4-bis (1,1-dimethylethyl)-(CAS) 2,4-di-tert-butylphenol	1.55
10.	36.00	Dodecanoic acid	1.07
11.	48.99	1,4-diaza-2,5-dioxo-3-isobutyl bicyclo[4.3.0] nonane	5.70
12.	49.60	Pyrrolo[1,2-a] pyrazine-1,4-dione, hexahydro-3-(2-methylpropyl)	4.63
13.	50.04	Cis-9-hexadecanoic acid	0.72
14.	50.68	Hexadecanoic acid	1.07
15.	56.42	Cis-vaccenic acid	1.07
16.	63.48	Pyrrolo [1,2-a] pyrazine-1,4-dione, hexahydro-3-(phenylmethyl)	1.21
**Postbiotic RS5 fermented in control medium**			
1.	3.09	Acetoin	3.68
2.	3.47	Propanoic acid, 2-hydroxy-methyl ester	34.68
3.	9.00	Lactic acid	32.53
4.	10.26	Pentanoic acid, 2-hydroxy-4-methyl-, methyl ester	0.92
5.	17.15	2-hydroxy-4-methylpentanoic acid	6.86
6.	17.60	2-3-dihydro-3,5-dihydroxy-6-methyl-4H-Pyran-4-one	5.18
7.	48.92	1,4-diaza-2,5-dioxo-3-isobutyl bicyclo[4.3.0] nonane	3.03
8.	49.92	Pyrrolo[1,2-a] pyrazine-1,4-dione, hexahydro-3-(2-methylpropyl)	3.63
9.	62.30	Pyrrolo [1,2-a] pyrazine-1,4-dione, hexahydro-3-(phenylmethyl)	9.48
**Postbiotic RS5 fermented in formulated medium**			
1.	3.10	Acetoin	1.82
2.	3.46	Propanoic acid, 2-hydroxy-methyl ester	2.94
3.	8.30	Lactic acid	92.53
4.	17.38	2-3-dihydro-3,5-dihydroxy-6-methyl-4H-Pyran-4-one	0.95
5.	48.94	1,4-diaza-2,5-dioxo-3-isobutyl bicycle [4.3.0] nonane	0.90
6.	49.55	Pyrrolo[1,2-a] pyrazine-1,4-dione, hexahydro-3-(2-methylpropyl)	0.86
**Postbiotic TL1 fermented in control medium**			
1.	3.09	Acetoin	4.22
2.	3.47	Propanoic acid, 2-hydroxy-methyl ester	22.98
3.	8.63	Lactic acid	55.33
4.	17.29	2-3-dihydro-3,5-dihydroxy-6-methyl-4H-Pyran-4-one	5.20
5.	48.85	1,4-diaza-2,5-dioxo-3-isobutyl bicycle [4.3.0] nonane	2.72
6.	49.47	Pyrrolo[1,2-a] pyrazine-1,4-dione, hexahydro-3-(2-methylpropyl)	3.03
7.	63.42	Pyrrolo [1,2-a] pyrazine-1,4-dione, hexahydro-3-(phenylmethyl)	6.53
**Postbiotic TL1 fermented in formulated medium**			
1.	3.11	Acetoin	0.66
2.	3.46	Propanoic acid, 2-hydroxy-methyl ester	3.82
3.	8.48	Lactic acid	92.04
4.	17.45	2-3-dihydro-3,5-dihydroxy-6-methyl-4H-Pyran-4-one	1.25
5.	48.98	1,4-diaza-2,5-dioxo-3-isobutyl bicycle [4.3.0] nonane	1.25
6.	49.59	Pyrrolo[1,2-a] pyrazine-1,4-dione, hexahydro-3-(2-methylpropyl)	0.98
**Postbiotic UL4 fermented in control medium**			
1.	2.70	Hydroxyacetone	0.39
2.	3.09	Acetoin	7.2
3.	3.47	Propanoic acid, 2-hydroxy-methyl ester	4.95
4.	8.63	Lactic acid	54.10
5.	10.24	Pentanoic acid, 2-hydroxy-4-methyl-, methyl ester	1.76
6.	17.06	2-hydroxy-4-methylpentanoic acid	8.67
7.	23.67	2-3-dihydro-3,5-dihydroxy-6-methyl-4H-Pyran-4-one	5.52
8.	48.90	1,4-diaza-2,5-dioxo-3-isobutyl bicycle [4.3.0] nonane	2.85
9.	48.92	Pyrrolo[1,2-a] pyrazine-1,4-dione, hexahydro-3-(2-methylpropyl)	4.06
10.	62.30	Pyrrolo [1,2-a] pyrazine-1,4-dione, hexahydro-3-(phenylmethyl)	10.09
**Postbiotic UL4 fermented in formulated medium**			
1.	3.10	Acetoin	2.96
2.	3.46	Propanoic acid, 2-hydroxy-methyl ester	2.14
3.	7.94	Lactic acid	87.94
4.	17.29	2-3-dihydro-3,5-dihydroxy-6-methyl-4H-Pyran-4-one	2.02
5.	48.98	1,4-diaza-2,5-dioxo-3-isobutyl bicycle [4.3.0] nonane	2.74
6.	49.59	Pyrrolo[1,2-a] pyrazine-1,4-dione, hexahydro-3-(2-methylpropyl)48.98	2.21

### Correlation Between Postbiotic Compositions and Functional Characteristics

The correlations between the six commonly found compounds in all the postbiotics with the inhibitory activity and antioxidant activity are shown in Table 6. A positive correlation (*P* < 0.05) was demonstrated between acetic acid production with inhibitory activity against *P. acidilactici* 446, *S. enterica* CS3, and *L. monocytogenes* LS55. A significant positive (*P* < 0.05) can also be detected between caproic acid production with the inhibitory activity against *E. coli* E-30, *L. monocytogenes* LS55, and VRE. The lactic acid production in the postbiotics had a significant positive correlation (*P* < 0.05) with inhibitory activity against *S. enterica* CS3 and HRS activity. Moreover, the propanoic acid, 2-hydroxy-methyl ester, 1,4-diaza-2,5-dioxo-3-isobutyl bicyclo [4.3.0] nonane, 2-3-dihydro-3,5-dihydroxy-6-methyl-4H-Pyran-4-one, and pyrrolo [1,2-a] pyrazine-1,4-dione, hexahydro-3-(2-methylpropyl) demonstrated a positive relationship with the HRS and RP activities, even though no significant correlation (*P* > 0.05) was detected between those compounds with all the inhibitory activity. However, the result revealed that acetoin exhibited a negative relationship with the inhibitory activity against *S. enterica* CS3 and HRS activity.

**Table 6:** Correlation test (R) between *L. plantarum* postbiotics composition and functional characteristics.

**Compounds**	***P. acidilactici* 446**	***E. coli* E-30**	***S. enterica* CS3**	***L. monocytogenes* LS55**	**VRE**	**HRS**	**RP**
Acetic acid	0.30^*^	−0.24	0.15^*^	0.34^*^	−0.36	0.12	0.16
Caproic acid	−0.27	0.40^**^	−0.03	0.44^**^	0.13^**^	−0.06	0.24
Acetoin	−0.47	−0.54	−0.66^*^	−0.09	−0.38	−0.62^*^	0.05
Propanoic acid, 2-hydroxy-methyl ester	−0.37	0.03	−0.52	−0.52	−0.17	0.68^*^	0.14
1,4-diaza-2,5-dioxo-3-isobutyl bicyclo [4.3.0] nonane	0.05	−0.18	0.05	0.02	0.07	0.53^*^	0.6^*^
Lactic acid	0.32	0.19	0.60^*^	0.46	0.33	0.84^***^	−0.32
2-3-dihydro-3,5-dihydroxy-6-methyl-4H-Pyran-4-one	0.05	−0.19	−0.28	−0.04	−0.18	0.76^**^	0.65^*^
Pyrrolo [1,2-a] pyrazine-1,4-dione, hexahydro-3-(2-methylpropyl)	−0.03	−0.19	−0.43	−0.11	−0.29	0.75^**^	0.60^*^

* Significant at (P < 0.05),

** significant at (P < 0.01),

*** *significant at (P < 0.001), and n = 36 samples per functional characteristic*.

## Discussion

### Inhibitory Activity of Postbiotics

The present study was conducted to elucidate the potential of the postbiotic metabolite produced by six strains of *L. plantarum* to replace AGPs, which have been used to curb the effects of inflammation in poultry. Generally, antibiotics have been widely used in livestock production as an antimicrobial against various pathogens such as *E. coli, Salmonella* sp., *Campylobacter* sp., *Pasteurella multocida, Mycoplasma* sp., *Clostridium perfringens*, and *Chlamydia psittaci* (43, 44). However, antibiotic abuse has accelerated the prevalence of antimicrobial-resistant bacteria and their transmissions to humans *via* the food chain (45, 46). In the present study, the growth of *L. plantarum* strains in formulated media produced postbiotic metabolites with enhanced biological functionalities, such as antimicrobial and antioxidant activities, through the production of the bioactive ingredients secreted extracellularly during the growth of producer cells. The higher inhibitory activity exerted by postbiotics produced by using formulated media could be attributed to the higher concentration of yeast extract that contains amino acids and growth factors (47). Thus, when incorporated in the formulated media, it had further promoted good bacterial cell growth and bacteriocin production as reported by Ooi et al. (31) and Abbasiliasi et al. (47). On top of that, the ingredients in MRS such as meat extract and peptone not only inhibit the growth but also reduce the antimicrobial activity of the bacteria (31, 48). For the carbon source, glucose has been proven to be the best ingredient for bacteriocin production due to its simple structure as compared to other carbon sources such as sucrose (31, 49).

Furthermore, the inhibitory activity of postbiotics was influenced by bacteriocin production and organic acid production. There are two classes of bacteriocin genes, namely, *plantaricin* EF and *plantaricin* W, which have been proven to be harbored by the novel *L. plantarum* I-UL4 (50), which are responsible for the production of plantaricin Ef and plantaricin W bacteriocins, respectively (51). The positive correlation (*P* < 0.05) between acetic acid, lactic acid, and caproic acid with inhibitory activities against various pathogens implying the organic acid production would further enhance the bacteriocin inhibitory activity of postbiotics. In addition, the secretion of a high concentration of organic acids and antioxidant compounds have been reported to contribute substantially to the inhibitory activity of postbiotic metabolites produced by various strains of *L. plantarum* (22, 52, 53).

In the livestock industry, inflammation due to bacterial infection is a common and profound challenge that caused an immense amount of loss and less profitable. The negative effects of bacterial infection can be avoided by using postbiotics since they exhibited a broad spectrum of antimicrobial activities against gram-positive and gram-negative pathogenic bacteria due to the presence of bacteriocin (52, 53) and organic acids (54, 55). The inhibitory activity exhibited by *L. plantarum* postbiotics in this experiment was in agreement with the previous reports (21, 22, 55) where the metabolite produced by *L. plantarum* strains exhibited antimicrobial effects on *P. acidilactici, S. enterica, E. coli*, VRE, and *L. monocytogenes*. This study also showed that postbiotic produced by using formulated media demonstrated a higher capability to inhibit the growth and proliferation of detrimental pathogenic bacteria, indicating that the postbiotic metabolites of *L. plantarum* strains were a potential alternative to AGP that exhibited probiotic effect without the inclusion of living cells (55).

### Antioxidant Activity of *L. plantarum* Postbiotics

The natural source of antioxidants has been used to counteract the implication due to oxidative stress and reactive oxygen species. Since oxidative stress antagonistically influences the occurrence of inflammation, cellular damage, and disease susceptibility in the body (56, 57), the postbiotic produced by *L. plantarum* has the potential to reduce the oxidation of proteins and lipids particularly through two mechanisms, namely, HRS and RP. Unlike other previous studies that reported on the DPPH and ABTS activities of *L. plantarum* (27, 58), two other assays were used in the current research to elucidate further the antioxidant activity of postbiotic produced by *L. plantarum*. HRS measured the scavenging activity of the most reactive radical, hydroxyl radical, which is related to lipid peroxidation and biomolecules of cells (59). In the present study, the HRS activity of the postbiotics was increased from 29 to 32% produced by using a control MRS medium to 31–37% when produced by using formulated media. Additionally, the postbiotic has an RP of 1.9–2.9 mg/L of ascorbic acid regardless of the fermentation medium used. The results obtained in this study were consistent with previous studies reported (60, 61) for having RP through the inhibition of the oxidation process by converting hydroperoxides to hydroxyoctadecadienoic acids and iron chelators in postbiotic due to the presence of various intracellular antioxidants such as pyrrole compounds in the postbiotic (62). Moreover, studies also revealed that the difference in the antioxidant activity of postbiotics depends on mechanisms such as the metal ion chelating ability, the antioxidant enzyme system, and the antioxidant metabolites present in the postbiotic (63). Therefore, postbiotic can be exploited as a supplement and feed additive to prevent inflammation due to oxidative stress-related diseases (55, 64). The antioxidant activity of postbiotics could be contributed by the formation of pyrrole and cyclic compounds as shown in Table 5.

The antioxidant activity exerted by *L. plantarum* helps to scavenge and inhibit free radicals against oxidation processes (35, 65). In the *in vitro* study conducted by Chen et al. (65), the fermentation of papaya juice by using *L. plantarum* produced higher antioxidant activities as compared to *L. acidophilus*. The in-feed supplementation of *L. plantarum* postbiotics has been documented to reduce the negative effect stimulated by hepatic injury in mice (66) and to improve glutathione peroxidase (GPX) in blood serum and ruminal fluid in post-weaning lamb (27), as well as to enhance the activities of total antioxidant capacity (T-AOC), catalase (CAT), and GPX while reducing alpha-1-acid glycoprotein (α1-AGP) and ceruloplasmin in broiler blood plasma (67). Therefore, *L. plantarum* postbiotic can be used as a promising natural source of antioxidants to reduce the effects of heat stress in animals. Moreover, the supplementation of *L. plantarum* has been shown to impede lipid peroxidation and suppressed oxidative stress in serum and liver induced by aflatoxin AFB1 (68).

### Organic Acid Production in *L. plantarum* Postbiotics

The SCFAs are known for their ability to down-regulate the production of pro-inflammatory cytokines expression and act on respective cells such as neutrophils and macrophages responsible for inducing inflammation (69). Besides acting as a regulator to intestinal health and mucous production to restrict the adhesion of pathogens on the epithelial cells (70–73), SCFAs are also responsible for the maintenance of the tight junction integrity to reduce the risk of intestinal inflammation (73), provide nutrient to hepatocytes (74), and influence hormonal regulation (75). Moreover, acetic acid also involves in the synthesis of cholesterol and fatty acids. Nevertheless, caproic acid and lactic acid are strong antimicrobial agents (76, 77) in decreasing the colonization of pathogenic bacteria in the gut while enhancing the digestibility of protein and mineral, respectively (68, 69). This agreed with the findings of this study where the high production of both VFAs detected in postbiotics inhibited the proliferation of pathogens. The high concentration of acetic acid retarded the growth of *P. acidilactici* 446, *S. enterica* CS3, and *L. monocytogenes* LS55, whereas the caproic acid exhibited the antimicrobial activity against *E. coli* E-30, *L. monocytogenes* LS55, and VRE.

All *L. plantarum* strains employed in this study produced lactic acid as the predominant metabolite product during the growth, which is a distinguishing characteristic of lactic acid bacteria (78). Lactic acid lowers the environmental pH and disrupts the process of nutrient absorption and energy uptake of bacteria by breaking down the cell membrane *via* lysis (75, 79). Hence, low pH conditions due to the presence of organic acids prohibited the proliferation and survival of pathogens. This supported the result that the higher secretion of lactic acid promoted antimicrobial action against the growth of salmonella. The high correlation between the lactic acid and HRS could indicate that lactic acid can be used as an antioxidant agent, which, in turn, can further enhance the antimicrobial activity of the postbiotic. As a result, even if there is a risk of pathogen infection, the development of the inflammation process will be altered by immunomodulating gut health through a microbial ecosystem (80). Lactic acid is well-reported for its antimicrobial activity against pathogenic and zoonotic bacteria in livestock farming such as *S. enteritidis, E. coli, L. monocytogenes, P. aeruginosa, S. aureus, E. faecalis*, and yeast (81–83). Besides, Van Thu et al. (21) had reported the production of acetic acid and lactic acid by the combination of postbiotics produced by various *L. plantarum* strains.

The presence of different concentrations of organic acids in the postbiotic produced by different strains of *L. plantarum* may be possibly due to the effects of fermentation media on heterofermentative biochemical pathways involving citrate metabolism (84), whereby the production of acetic acid was lower than the lactic acid as shown by various *L. plantarum strains* used in this study. Despite the fact that there is a paucity of knowledge on the effects of fermentation media on the expression of gene related to organic acid production in each strain of bacteria, this study provided the fundamental knowledge on the influence of different fermentation media and bacteria strains on the production of organic acid in postbiotics.

### Identification of Volatile Compounds in Postbiotics Using GC-MS

The production of compounds such as organic acids, acetoin, intermediary compounds, and pyrrole compounds are commonly found in the postbiotic after bacterial fermentation. Acetoin, for example, is closely related to food flavor, and it is also involved in the production of the amino acid (85). Furthermore, previous studies documented that cyclic and pyrrole compounds such as 1,4-diaza-2,5-dioxo-3-isobutyl bicyclo [4.3.0] nonane, 2-3-dihydro-3,5-dihydroxy-6-methyl-4H-Pyran-4-one, and pyrrolo [1,2-a] pyrazine-1,4-dione, hexahydro-3-(2-methylpropyl) are strong antioxidant and antifungal agents (86–90). Therefore, the secretion of pyrrole and cyclic compounds in the postbiotics enhanced the antimicrobial activity against various pathogenic bacteria and the ability to inhibit or prevent the oxidation process.

Several studies have revealed that viable probiotic cells and the corresponding postbiotic of *L. plantarum* possessed anti-inflammatory properties. Toshimitsu et al. (91) reported that *L. plantarum* induced IL-10 production and ameliorated the metabolic effect of type 2 diabetes in mice. Furthermore, probiotic *L. plantarum* has been demonstrated to improve the gut barrier function and up-regulated inflammatory mediators when induced with lipopolysaccharides (92), and posed an immunomodulatory effect on intestinal epithelial and mononuclear cells (93). Similarly, the supplementation of postbiotics produced by *L. plantarum* strains to livestock ameliorated the inflammation incidence by up-regulated anti-inflammatory cytokines and down-regulated pro-inflammatory cytokines, while modulating immune response to prevent dysbiosis (21, 24, 28, 94). Moreover, postbiotic *L. plantarum* strains exerted a cytotoxicity effect on various cancer cells without causing hemolysis on erythrocytes obtained from humans and several animal species (95). This implies that the postbiotics of *L. plantarum* strains contain beneficial compounds such as organic acids, pyrrole compounds, and other intermediary compounds, which contributed to high antioxidant, antibacterial, anti-inflammatory, antimicrobial, and anticancer properties (96, 97). Besides that, this agreed with the finding of a positive correlation between the secretion of acetic acid, caproic acid, and various pyrrole and cyclic compounds detected in all the postbiotics with inhibitory activity and antioxidant activity. In other words, enhancement of postbiotic compositions would greatly enhance its functional characteristics.

## Conclusion

In conclusion, the effect of formulated media on the compositions and functional characteristics of postbiotics produced by the six strains of *L. plantarum* was elucidated in this study. The composition and functional characteristics of postbiotics were strain-dependent, whereby postbiotic RG11, RI11, and RS5 exhibited higher inhibitory and antioxidant activities, which contained a higher concentration of acetic acid, caproic acid, and lactic acid. The current study also revealed that fermentation media were a prior important factor that exerted a great impact on the functionality characteristics of postbiotics such as the production of pyrrole compounds and other intermediary compounds, which contributed to high antioxidant, antibacterial, anti-inflammatory, antimicrobial, and anticancer properties. The postbiotics of *L. plantarum* strains produced by using a formulated medium warrant promising potential to be applied as a natural bioactive growth promoter for the livestock industry.

## Data Availability Statement

The raw data supporting the conclusions of this article will be made available by the authors, without undue reservation.

## Author Contributions

HMC, TCL, HLF, and EL took part in designing the experiment. HMC and NEA performed the experiment and data analysis, and wrote the paper. All authors participated in reading, provided a critical review, and approved the final manuscript.

## Conflict of Interest

The authors declare that the research was conducted in the absence of any commercial or financial relationships that could be construed as a potential conflict of interest.
